# Association Between Serum Uric Acid Level and Carotid Atherosclerosis and Metabolic Syndrome in Patients With Type 2 Diabetes Mellitus

**DOI:** 10.3389/fendo.2022.890305

**Published:** 2022-06-13

**Authors:** Wei Li, Yan Wang, Shengrong Ouyang, Mengdi Li, Rui Liu, Yuqi Zhang, Xiaojun Liu, Tianfang Li, Shengyun Liu

**Affiliations:** ^1^ Department of Rheumatology, The First Affiliated Hospital of Zhengzhou University, Zhengzhou, China; ^2^ Department of Respiratory and Critical Care Medicine, The First Affiliated Hospital of Zhengzhou University, Zhengzhou, China; ^3^ Department of Biochemistry and Immunology, Capital Institute of Pediatrics, Beijing, China

**Keywords:** uric acid, metabolic syndrome, carotid atherosclerosis, intima-media thickness, type 2 diabetes mellitus

## Abstract

**Background:**

Serum uric acid (SUA) is associated with many cardiovascular risk factors, such as metabolic syndrome (MetS) and subclinical atherosclerosis. However, the relationship of SUA with carotid atherosclerosis remains controversial. We aimed to investigate whether elevated SUA levels are associated with a high risk of carotid atherosclerosis and MetS in patients with type 2 diabetes mellitus (T2DM).

**Methods:**

This cross-sectional study was performed with a sample of 1,947 hospitalized patients with T2DM. Carotid intima-media thickness and carotid artery plaques were measured *via* Doppler ultrasound.

**Results:**

Uric acid levels were negatively associated with HbA1C, eGFR, and HDL-C (all P < 0.001) and positively associated with WBC, BMI, ACR, creatinine, total cholesterol, triglycerides, LDL-C, systolic blood pressure, and diastolic blood pressure (all P < 0.001). After adjusting for multiple potential confounders, the risks were substantially higher for MetS in the highest quartile of SUA levels (odds ratio: 2.91, 95% confidence interval: 1.54–5.51, P = 0.003 for trend) than in the lowest quartile of SUA levels. Furthermore, a significant increase was observed in the prevalence of overweight/obesity, hypertension, and dyslipidemia across the SUA quartiles independent of confounders. However, no significant association was found between SUA quartile with the presence of carotid atherosclerosis.

**Conclusions:**

In patients with T2DM, SUA levels were closely associated with MetS and its components but not with carotid atherosclerosis.

## Background

Accumulating epidemiological and clinical evidence demonstrated that serum uric acid (SUA) levels are strongly associated with many cardiovascular risk factors, such as obesity, hypertension, hyperlipidemia, diabetes, metabolic syndrome (MetS), and subclinical atherosclerosis (1–3). Among these risk factors, the associations of SUA with atherosclerosis and MetS have been observed in several studies in both general and type 2 diabetic mellitus (T2DM) populations (4, 5).

Uric acid is the end metabolic product of purine in humans. Hyperuricemia can lead to various diseases, and it is most notably involved in the pathogenesis of gouty arthritis (3). Previous studies have suggested that hyperuricemia is a risk factor for cardiovascular disease (CVD) in the general population (6, 7). Ultrasound of the carotid artery to identify carotid intima-media thickness (c-IMT) and carotid artery plaques (CAP) can predict the risk of CVD. As a surrogate of atherosclerosis diseases, CAP accounts for approximately a fifth of the risk of stroke and coronary artery diseases (8, 9). However, the associations between SUA concentration and carotid atherosclerosis, as reflected by c-IMT and CAP, have previously been studied but produced conflicting results. A population-based cross-sectional survey demonstrated that SUA level is associated with MetS and an independent risk factor for carotid atherosclerosis in patients with T2DM (4). Another study showed that SUA levels are closely associated with hypertension and MetS but not with atherosclerosis in people with diabetes (10). Some authors considered that the role of uric acid in atherosclerosis might be attributed to other cardiovascular risk factors, such as hypertension, obesity, MetS, and chronic kidney disease (11, 12). Furthermore, uric acid-induced inflammatory pathway may play an important role in the pathogenesis of MetS, increased uric acid levels have been founded in inflammatory conditions (13), the role of inflammation in the association between SUA and carotid atherosclerosis should be examined.

Although previous studies have showed that the independence of relationship between SUA and MetS and atherosclerosis. However, few studies have examined the relationship between SUA and components of MetS, as well as the association between SUA and carotid atherosclerosis in patients with T2DM. Therefore, this study aimed to investigate the association between SUA level and MetS and carotid atherosclerosis in T2DM populations.

## Methods

### Study Subjects

This cross-sectional study evaluated the prevalence of MetS and carotid atherosclerosis in patients with T2DM aged over 18 who were hospitalized at the First Affiliated Hospital of Zhengzhou University from January 2018 to December 2020. Patients who were taking any drug that might interfere with uric acid metabolism, such as allopurinol, furosemide, and thiazides, etc, were excluded (N = 85). Patients who did not undergo carotid ultrasound examination and without complete clinical and SUA data were also excluded (N = 368). In total, 1,947 patients, including 1,335 males, were included in the final analyses. All patients underwent an interview and provided a history of hypertension, CVD, duration of diabetes, use of lipid-lowering drugs and antihypertensive agents, alcohol consumption, and smoking habits. Body mass index (BMI) was calculated by body weight (kg) divided by height squared (m^2^). Blood pressure was measured by using an automatic blood-pressure meter after the participants sat for at least 10 min. The average of three measurements was recorded for further analysis. This study was approved by the Institution Review Board of the First Affiliated Hospital of Zhengzhou University.

### Laboratory Measurements

The patients were asked to fast overnight, and then blood samples were obtained for further analysis. Total cholesterol (TC), high-density lipoprotein cholesterol (HDL-C), low-density lipoprotein cholesterol (LDL-C), triglycerides (TG), fasting blood glucose (FBG), insulin, SUA, creatinine, white blood cells (WBC), and C-reactive protein (CRP) were measured. HbA1c level was measured *via* high-performance liquid chromatography. A sterile, random-spot urine sample was used to measure the albumin/creatinine ratio (ACR). The estimated glomerular filtration rate (eGFR) was calculated using the simplified Modification of Diet in Renal Disease formula: eGFR = 186.3 × (serum creatinine)^−1.154^ × (age)^−0.203^ × (0.742 if female).

### Assessment of CAP and c-IMT

Carotid ultrasonography was performed using a color Doppler ultrasonic diagnostic instrument. Trained and certified sonographers conducted the examination. c-IMT was determined at the point approximately 1.5 cm away from the distal part of the bifurcation of common carotid artery. c-IMT was calculated as the mean of the intima-media thicknesses of the left and right common carotid arteries. CAP was defined as a focal region with a thickness of ≥1.5 mm as measured from the media adventitia interface to the lumen–intima interface or as the presence of focal wall thickening that was at least 50% greater than that of the surrounding vessel wall.

### Definition of MetS

MetS was defined on the basis of the updated National Cholesterol Education Program Adult Treatment Panel III criteria for Asian-Americans as presenting at least three of the following components: 1) waist circumference 90 cm or greater in men or 80 cm or greater in women; 2) TG 1.7 mmol/L or greater; 3) HDL-C less than 1.03 mmol/L in men or less than 1.30 mmol/L in women; 4) blood pressure 130/85 mmHg or greater or current use of antihypertensive medications; or 5) fasting plasma glucose 5.6 mmol/L or greater or previously diagnosed with T2DM or on oral antidiabetic agents or insulin (14).

### Statistical Analysis

Continuous variables were checked for the normal distribution using Kolmogorov–Smirnov statistics. Normally distributed data were expressed as means ± SD, whereas variables with a skewed distribution were reported as median (interquartile range, IQR). Categorical variables were represented by frequency and percentage. Kruskal–Wallis test was used to analyze groups differences for continuous variables, and Chi-square test was used for categorical variables. Spearman correlation coefficients between SUA and metabolic features were calculated by partial correlation analysis on ranks. Multivariate logistic regression models were used to estimate the odds ratios (ORs) for CAP and MetS according to SUA quartiles. Potential confounding variables, including age, gender, smoking, alcohol drinking, duration of diabetes, self-reported CVD, eGFR, FBG, HbA1C, CRP, and BMI, were controlled in the regression models. All statistical analyses were performed using SPSS version 26.0 (SPSS, Chicago, IL, USA). P < 0.05 was considered statistically significant.

## Results

### Characteristics of the Participants According to SUA Quartiles

We identified 1,947 patients with T2DM with a mean age of 49.6 ± 11.9 years. The population studied herein was stratified into quartiles according to SUA levels. The baseline demographic and medical characteristics for SUA quartiles are provided in **Table 1**. The cut-off SUA values for Q1, Q2, Q3, and Q4 were <242, 242–293, 293–353, and ≥353 µmol/L, respectively. When analyzed by quartiles of SUA levels, the patients with higher uric acid levels were more likely to be male, smokers, drinker, and younger (all P < 0.001). With respect to metabolic parameters, the patients in the higher uric acid quartiles exhibited higher levels of systolic blood pressure (SBP) and diastolic blood pressure (DBP), BMI, CRP, creatinine, insulin, ACR, TG, and TC than those in the lower uric acid quartiles (all P < 0.05). By contrast, the patients with higher uric acid levels displayed shorter duration of diabetes and lower levels of HbA1C and HDL-C than those with lower uric acid levels (all P < 0.05). However, no difference in c-IMT and CAP was observed between the SUA quartile groups.

**Table 1:** Characteristics of study participants according to uric acid quartiles.

	Q1	Q2	Q3	Q4	P value
	N = 485 (< 242)	N = 485 (242-293)	N = 489 (293-353)	N = 488 (≥ 353)	
Male, n (%)	213 (43.9)	299 (61.6)	384 (78.5)	439 (90)	0
Age, years	54 (47–62)	53 (45–60)	50 (42–58)	46 (35–54)	0
DD, years	5 (1–10)	5 (0.9–10)	5 (2–11)	3 (0.3–10)	0.049
CVD, n (%)	46 (9.5)	60 (12.4)	40 (8.2)	41 (8.4)	0.1
Hypertension, n (%)	171 (35.3)	221 (45.6)	206 (42.1)	252 (51.6)	0
Smoking, n (%)	75 (15.5)	124 (25.6)	141 (28.8)	157 (32.2)	0
Alcohol, n (%)	60 (12.4)	102 (21)	125 (25.6)	129 (26.4)	0
BMI, kg/m^2^	24 (22–26.2)	24.4 (22.9–27.7)	26 (23.9–28)	26.9 (24.7–30)	0
HbA1C, %	9 (7.5–10.6)	8.6 (7.3–10.2)	8.5 (7.4–10.1)	8.1 (6.7–10.4)	0
FBG, mmol/L	7.8 (6.2–10.6)	7.8 (6.0–10.0)	7.8 (6.5–10.3)	7.4 (6.0–10.8)	0.199
Insulin, μU/mL	4.54 (2.1–8.1)	4.9 (2.5–8.6)	5.5 (2.8–11.1)	7.0 (3.7–13.8)	0
CRP, mg/L	0.85 (0.49–2.16)	0.96 (0.51–2.54)	1.09 (0.53–2.43)	1.78 (0.86–3.82)	0
WBC	6 (5.1–7.4)	6.1 (5.1–7.4)	6.37 (5.5–7.6)	6.6 (5.6–8.0)	0
ACR, mg/mmoL	0.67 (0.38–1.43)	0.68 (0.4–2.19)	0.8 (0.34–3.74)	1.29 (0.47–5.82)	0
Creatinine, μmol/L	54 (47.7–65.5)	61 (53–69.7)	67 (58–76.4)	71 (62–81)	0
SUA, μmol/L	206 (181–223)	268 (256–282)	319 (305–337)	410 (381–459)	0
eGFR, ml/min/1.73^2^	105.7 (99.2–115.0)	104.3 (98.1–113.9)	105.3 (94.6–114.2)	106.1 (90.9–118.8)	0.418
SBP, mmHg	129 (120–143)	131 (120–141)	135 (126–145)	133 (126–143)	0.004
DBP, mmHg	80 (73–87)	81 (74–89)	84 (79–92)	85 (79–92)	0
MetS, n (%)	277 (57.1)	353 (72.8)	392 (80.2)	429 (87.9)	0
CAP, n (%)	275 (56.7)	258 (53.2)	244 (49.9)	238 (48.8)	0.058
c-IMT, mm	1.3 (1.1–1.3)	1.2 (1.1–1.3)	1.2 (1.1–1.3)	1.2 (1.1–1.3)	0.179
TC, mmol/L	4.43 (3.8–5.1)	4.34 (3.6–5.2)	4.38 (3.8–5.1)	4.44 (3.8–5.2)	0.012
TG, mmol/L	1.26 (0.9–2.0)	1.68 (1.1–2.6)	1.88 (1.2–3.0)	2.09 (1.4–3.9)	0
HDL-C, mmol/L	1.13 (0.9–1.4)	1.03 (0.9–1.3)	0.99 (0.8–1.2)	0.95 (0.8–1.2)	0
LDL-C, mmol/L	2.69 (2.1–3.3)	2.51 (1.9–3.5)	2.61 (2.0–3.2)	2.48 (1.8–3.1)	0.189

ACR, albumin/creatinine ratio; BMI, body mass index; c-IMT, carotid intima-media thickness; CAP, carotid artery plaques; CRP, C-reactive protein; DBP, diastolic blood pressure; DD, duration of diabetes; eGFR, estimated glomerular filtration; FBG, fasting blood glucose; HDL-C, high-density lipoprotein cholesterol; LDL-C, low-density lipoprotein cholesterol; MetS, metabolic syndrome; SBP, systolic blood pressure; SUA, serum uric acid; TC, total cholesterol; TG, total triglycerides; WBC, white blood cells.

### Correlation Between SUA and Other Parameters

**Figure 1:**
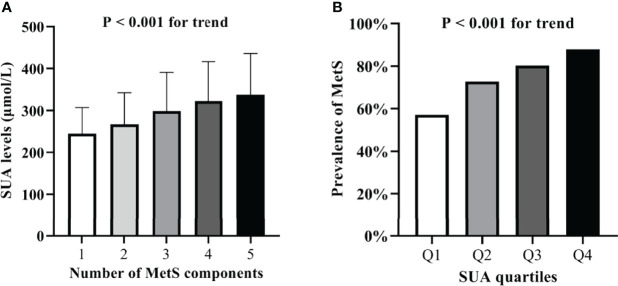
**(A)** Serum uric acid levels according to the number of MetS components. **(B)** Comparison of the prevalence of MetS among the four SUA quartile groups.

Partial correlation analysis revealed close correlation between SUA levels and BMI, HbA1C, ACR, creatinine, eGFR, SBP, DBP, TC, TG, and HDL-C among various metabolic features after adjusting for age, gender, and duration of diabetes (**Table 2**). Remarkably, SUA levels gradually increased with increasing number of MetS components. The mean values of SUA concentrations significantly increased for those with one, two, three, four, and five components of MetS; the mean values were 244.3 ± 62.7, 266.9 ± 75.5, 298.7 ± 92.5, 322.2 ± 94.3, and 337.7 ± 98.2 μmol/L, respectively (P < 0.001, **Figure 1A**). Furthermore, the prevalence of MetS was higher with increasing SUA quartiles; 57.10%, 72.80%, 80.20%, and 87.90% for Q1, Q2, Q3, and Q4, respectively (P < 0.001 for trend, **Figure 1B**).

**Table 2:** Correlation between SUA and other parameters.

Variable	Correlation coefficient	P value
BMI, kg/m^2^	0.325	0
CRP, mg/L	0.019	0.514
WBC	0.129	0
HbA1C, %	-0.173	0
ACR, mg/mmoL	0.152	0
FBG, mmol/L	-0.044	0.056
Creatinine, μmol/L	0.292	0
eGFR, ml/min/1.73^2^	-0.33	0
TC, mmol/L	0.105	0
TG, mmol/L	0.184	0
HDL-C, mmol/L	-0.148	0
LDL-C, mmol/L	0.013	0.56
SBP, mmHg	0.092	0
DBP, mmHg	0.099	0
c-IMT, mm	-0.06	0.355

All correlation coefficients were calculated after adjustment for age, gender, and diabetes duration.

### Comparison of MetS and CAP Between the SUA Quartile Groups

As presented in **Table 3**, the OR for MetS was higher with increasing SUA quartiles after adjusting for age and gender (OR: 5.132, 95% CI: 3.63–7.25, P < 0.001 for trend). In the highest uric acid quartile, the OR was 2.91 (95% CI: 1.54–5.51, P = 0.003 for trend) for MetS after further adjusting for alcohol drinking, smoking, duration of diabetes, self-reported CVD, BMI, CRP, HbA1C, FBG, eGFR, TC, and LDL-C. A comparison of CAP between the SUA quartile groups after adjusting for multiple potential confounders is given in **Table 3**. However, no significant differences were observed in the prevalence of CAP across the four groups.

**Table 3:** Adjusted ORs and 95% CIs for MetS and CAP according to SUA quartiles.

	Q1	Q2	Q3	Q4	P value
	OR (95% CI)				
CAP					
Model 1	1	0.85 (0.64–1.14)	0.90 (0.67–1.21)	1.07 (0.78–1.47)	0.408
Model 2	1	0.77 (0.58–1.04)	0.82 (0.60–1.11)	0.90 (0.65–1.25)	0.332
Model 3	1	0.80 (0.59–1.08)	0.85 (0.62–1.16)	0.97 (0.69–1.37)	0.38
Model 4	1	0.82 (0.54–1.24)	0.87 (0.56–1.35)	1.07 (0.65–1.76)	0.559
MetS					
Model 1*	1	2.00 (1.52–2.63)	2.94 (2.18–3.96)	5.13 (3.63–7.25)	0
Model 2*	1	1.96 (1.49–2.58)	2.94 (2.18–3.97)	5.17 (3.65–7.32)	0
Model 3*	1	1.41 (1.01–1.97)	1.69 (1.18–2.42)	2.66 (1.77–4.01)	0
Model 4*	1	2.01 (1.24–3.25)	1.99 (1.17–3.37)	2.91 (1.54–5.51)	0.003

Model 1 adjusted for age and gender.

Model 2 further adjusted for alcohol drinking, smoking, duration of diabetes, hypertension, and history of CVD.

Model 3 further adjusted for BMI.

Model 4 further adjusted for eGFR, CRP, FBG, HbA1C, TG, TC, HDL-C, and LDL-C.

Model 1* adjusted for age and gender.

Model 2* further adjusted for alcohol drinking, smoking, duration of diabetes, and history of CVD.

Model 3* further adjusted for BMI.

Model 4* further adjusted for eGFR, CRP, FBG, HbA1C, TC, and LDL-C.

### Association Between SUA Quartiles and Other MetS Components

The associations of the SUA quartiles with different MetS components in all patients with T2DM are summarized in **Table 4**. After controlling for multiple confounding factors, the SUA quartiles were found to be independently associated with increased prevalence of hypertension in T2DM populations. The OR for hypertension increased with increasing SUA quartiles. In the highest SUA quartile, the OR was 2.13 (95% CI: 1.34–3.38, P = 0.015) for hypertension. Similarly, in the highest SUA quartile, the OR was 3.45 (95% CI: 2.25–5.28, P < 0.001) for overweight/obesity (BMI ≥ 25 kg/m^2^). Dyslipidemia was defined as TC ≥ 6.2 mmol/L, or TG = 1.7 mmol/L or greater, or HDL-C < 1.03 mmol/L in men or < 1.30 mmol/L in women. In our patients with T2DM, after controlling for confounding factors, the SUA quartiles were still independently associated with increased prevalence of dyslipidemia. In the highest SUA quartile, the OR for dyslipidemia (TG ≥ 1.7 mmol/L) was 2.71 (95% CI: 1.73–4.25, P < 0.001), and the OR for dyslipidemia (TC ≥ 6.2 mmol/L) was 2.51 (95% CI: 1.17–5.38, P = 0.013). However, no significant differences were found in the prevalence of dyslipidemia (HDL-C abnormality) across the four SUA quartile groups.

**Table 4:** Association of SUA quartiles with MetS components.

	Q1	Q2	Q3	Q4	P value
	OR (95% CI)				
Overweight/Obesity	1	1.56 (1.08–2.23)	2.56 (1.74–3.76)	3.45 (2.25–5.28)	0
Hypertension	1	1.40 (0.94–2.08)	1.41 (0.93–2.15)	2.13 (1.34–3.38)	0.015
Dyslipidemia (TG)	1	1.61 (1.09–2.37)	2.56 (1.70–3.85)	2.71 (1.73–4.25)	0
Dyslipidemia (TC)	1	0.71 (0.32–1.57)	1.41 (0.68–2.94)	2.51 (1.17–5.38)	0.013
Dyslipidemia (HDL-C)	1	1.45 (0.99–2.12)	1.36 (0.91–2.03)	1.52 (0.98–2.37)	0.195

Adjusted for age, sex, alcohol drinking, smoking, duration of diabetes, self-reported CVD, BMI, CRP, HbA1C, FBG, eGFR.

## Discussion

SUA levels were strongly associated with the presence of MetS but not with the presence of carotid atherosclerosis in the patients with T2DM. In the highest SUA quartile, the ORs were 2.91 (95% CI: 1.54–5.51) for MetS after further adjusting for other atherosclerotic risk factors, such as age, gender, BMI, eGFR, and other lipid and glycemic parameters. However, SUA quartile was not associated with the presence of carotid artery plaque. Furthermore, the prevalence of hypertension, dyslipidemia, and overweight/obesity, substantially increased across the SUA quartiles independent of potential confounders.

Many studies have evaluated the associations between uric acid and MetS (15, 16). Consistent with the results of previous studies (4, 10), we observed a strong relationship between SUA and MetS. The ORs were substantially higher for MetS (OR: 2.91, 95% CI 1.54–5.51, P = 0.003 for trend) in the highest SUA quartile than those in the lowest SUA quartile. We also observed that SUA concentration increased with the number of MetS components (P < 0.001 for trend). Moreover, we found that the patients in the higher SUA quartiles had greater numbers of MetS components than the patients in the lower SUA quartiles. However, the underlying mechanisms of the association between SUA and MetS remain largely unknown. Previous studies indicated that hyperuricemia may be partially responsible for the inflammatory process in adipose tissues and vascular endothelial cells that will lead to a chronic low-grade inflammation and insulin resistance in subjects with MetS (17, 18). A recent study showed that higher SUA levels present higher levels of CRP and increased serum levels of inflammatory cytokines, indicating that SUA may be an inductor of subclinical inflammation (13). Consistent with this supposition, we observed that the acute phase biomarkers, including WBC and CRP levels, gradually increased with SUA quartiles (P < 0.001). Therefore, given that low-grade inflammation and insulin resistance are two major risks factors for MetS, uric acid-induced inflammatory pathway may play an important role in the pathogenesis of MetS. Interestingly, previous studies have demonstrated that reducing uric acid substantially improves systemic inflammation, endothelial function, and peripheral vasodilator capacity (19, 20). Results of studies from the URRAH database further strengthen the role of uric acid in CVD, the identified cut-off values support clinicians consider uric acid as an additional cardiovascular risk factor (21). Thus, SUA may be a promising candidate for risk assessment and a potential intervention target for MetS and CVD (17).

Numerous studies have demonstrated that SUA levels are independently associated with the presence of hypertension (22, 23). In previous retrospective cohort study showed that elevated SUA levels could be associated with poor blood pressure and diabetic control (24, 25). A recent study suggested that uric acid has independent effects on the development of hypertension and MetS but is not an independent risk factor for atherosclerosis in patients with T2DM (10). Our results were in agreement with these findings. The ORs for hypertension increased with increasing SUA quartiles. In the highest SUA quartile, the OR was 2.13 (95% CI: 1.34–3.38, P < 0.015) for hypertension. Experimental studies have also suggested the potential roles of uric acid in the pathogenesis of hypertension. The mechanism by which uric acid causes hypertension may be due to the inhibition of the release of endothelial nitric oxide and the activation of the rennin–angiotensin system, which lead to oxidative stress, endothelial dysfunction, and smooth muscle cell proliferation, and ultimately to elevated blood pressure (26, 27).

In the past decades, several studies have assessed the relationship between SUA and the components of MetS in different selected populations. Multiple clinical and epidemiological studies have also demonstrated the strong association among SUA and obesity, hypertension, and MetS (28, 29). A previous study reported that the dyslipidemia components of serum TC, TG, and LDL-C levels are positively associated with SUA levels, whereas serum HDL-C levels are inversely related with SUA (30). Our analysis was consistent with these findings. A notable increase in the risk of overweight/obesity, hypertension, and dyslipidemia was observed across the SUA quartiles after adjusting for known potential confounders. However, the issue of whether hyperuricemia is a downstream result of MetS, or it may play an upstream role in MetS development remains unclear. The Mendelian randomization (MR) technique enables their use as instrumental variables for testing causality by exploiting the random distribution of genetic variants (31). An MR investigation suggested that SUA may augment the risk of MetS by increasing blood pressure and TC levels and lowering HDL-C levels but not by accumulating fat or hyperglycemia. Obesity may be a causal agent for all the components of MetS, including hyperuricemia (32). Meanwhile, a recent study of obese adults indicated that SUA has no apparent association with hypertension, dyslipidemia, T2DM, and cardiovascular events (33). Thus, further complementary studies on the causal relationship and the potential mechanism between SUA and components of MetS are warranted.

However, the results regarding the associations of SUA with atherosclerosis and CVD from different studies remains controversial (4, 34). Several clinical studies have reported that elevated SUA levels are independent predictors of atherosclerosis, CVD, and mortality in different populations (5). However, other epidemiological studies have failed to confirm such associations and argued that these relationship are not causal but rather a result of a coexistence with other cardiovascular risk factors, such as obesity, MetS, and chronic kidney disease (10, 11). A recent study indicated that SUA appears to be strongly correlated with c-IMT but not with the prevalence of carotid plaques or aortic stiffness (26). The present study did not observe any association between SUA and c-IMT and carotid atherosclerotic plaques in T2DM populations despite adjusting for all known confounders. Thus, these results demonstrated that SUA may not be a risk factor for CAP and that the association between SUA and carotid atherosclerosis is not truly independent. Additionally, the methodological differences and the different characteristics of study populations might account for the discrepancies reported in the literature and by the present study. Therefore, given that the SUA levels were strongly associated with MetS and its components, SUA may play an indirect role in the pathogenesis of atherosclerosis *via* other CVD factors, such as obesity, hypertension, dyslipidemia, and MetS in some selected populations.

Owing to the cross-sectional nature of this study, it has several limitations. The mechanisms underlying these associations remain to be explored. The study findings are inherently limited in the ability to eliminate causal effect relationships between SUA and MetS. The participants of the present study were Chinese patients with T2DM. Therefore, the present results might not be representative of the general population. Moreover, many cardiovascular risk factors, such as glucose and lipid metabolic disorders, can accumulate in patients with T2DM and might affect the role of SUA in the development of carotid atherosclerosis and CVD.

## Conclusion

In summary, the findings of this study strongly suggested that SUA has independent association with the prevalence of hypertension, obesity, dyslipidemia and MetS but not with carotid atherosclerosis in T2DM populations. Our findings demonstrated that the role of uric acid in atherosclerosis might be attributed to other cardiovascular risk factors, such as MetS and its components. Prospective studies are required to clarify further the causal associations of SUA with MetS and carotid atherosclerosis in patients with T2DM.

## Data Availability Statement

The original contributions presented in the study are included in the article/supplementary material. Further inquiries can be directed to the corresponding authors.

## Ethics Statement

The studies involving human participants were reviewed and approved by Institution Review Board of the First Affiliated Hospital of Zhengzhou University. Written informed consent for participation was not required for this study in accordance with the national legislation and the institutional requirements.

## Author Contributions

WL, YW, TL, XL, and SL contributed to the conception and design of the study. WL, YW, SL, YZ, ML, and RL recruited the subjects and supervised the study. WL, YW and SO analyzed the data. WL and SL wrote the initial draft of the paper. WL, YW, TL, XL, and SL contributed to the writing, reviewing, and revising of the manuscript. All authors contributed to the article and approved the submitted version.

## Funding

WL is funded by the National Natural Science Foundation of China (82000831).

## Conflict of Interest

The authors declare that the research was conducted in the absence of any commercial or financial relationships that could be construed as a potential conflict of interest.

## Publisher’s Note

All claims expressed in this article are solely those of the authors and do not necessarily represent those of their affiliated organizations, or those of the publisher, the editors and the reviewers. Any product that may be evaluated in this article, or claim that may be made by its manufacturer, is not guaranteed or endorsed by the publisher.

## Abbreviations

ACR, Albumin/creatinine ratio; BMI, Body mass index; CAP, Carotid artery plaques; CI, Confidence interval; c-IMT, Carotid intima-media thickness; CVD, Cardiovascular disease; DBP, Diastolic blood pressure; eGFR, Estimated glomerular filtration rate; FBG, Fasting plasma glucose; HbA1c, Glycosylated hemoglobin; HDL-C, High-density lipoprotein cholesterol; LDL-C, Low-density lipoprotein cholesterol; MetS, metabolic syndrome; OR, Odds ratio; SBP, Systolic blood pressure; TC, Total cholesterol; TG, Triglyceride; T2DM, Type 2 diabetes mellitus; SUA, Serum uric acid.
